# Embedding pragmatic clinical trials in graduate medical education: Lessons from implementation

**DOI:** 10.1017/cts.2026.10687

**Published:** 2026-01-28

**Authors:** David C. Newton, Todd W. Rice, Robert Edward Freundlich

**Affiliations:** 1 Anesthesiology, Vanderbilt University Medical Center, USA; 2 Allergy, Pulmonary and Critical Care Medicine, Vanderbilt University Medical Center, USA

**Keywords:** Ethics, graduate medical education, pragmatic, informed consent, pragmatic trial

Pragmatic clinical trials (PCTs) provide high-quality, generalizable evidence at lower cost and often faster pace than traditional randomized controlled trials [1]. However, it can be challenging to embed interventions into complex workflows and satisfy the regulatory elements inherent in clinical research. We propose that graduate medical education (GME) trainees can address these design challenges, offering insights from a large, randomized PCT embedded within a resident-led care team. These lessons may allow researchers to thoughtfully incorporate GME trainees into future pragmatic studies.

At our institution, a PCT of perioperative ketamine infusion versus placebo was integrated into the workflow of a resident-led perioperative team. Study participants were enrolled using a modified consent completed by anesthesiology residents alongside routine clinical evaluation and procedural consent [2]. Residents were provided a standardized script to guide conversation, and patients could complete a short form consent if they wished to participate. This structure was designed in collaboration with the local Institutional Review Board (IRB) and intended to be innovative. Using this approach, residents enrolled 1544 patients as part of their routine clinical duties, demonstrating its enrollment efficacy and offering a template for future pragmatic study design.

We also wanted to understand how trainees perceived their participation in pragmatic research. We surveyed current and former residents who enrolled patients into this PCT to assess residents’ (1) attitudes toward pragmatic trials within their clinical residency education and (2) comfort with the ethics of pragmatic research, particularly with an abbreviated informed consent process. The survey (Appendix) was approved by the local IRB and included anesthesiology residents who completed at least one year of their training during active trial enrollment (4/12/2021–1/26/2024), creating a total sample of 91 residents.

After six weeks, 50/91 residents (55%) had responded. Their opinions on the appropriateness of their involvement were mixed, with 30/50 (60%) of survey participants reporting that the use of the resident workforce was either absolutely or somewhat appropriate to consent and enroll patients, 11/50 (22%) reported it as somewhat inappropriate, and 9/50 (18%) were neutral. When asked whether they would recommend this pragmatic design be used to answer future research questions in their field, 21/50 (42%) of survey participants would recommend it, 15/50 (30%) would not recommend it, and 14/50 (28%) were neutral.

We also asked trainees about their perspectives using the alteration of informed consent, a process permitted when research involves no more than minimal risk, both treatments would be included in the standard of care, and the alteration would not adversely affect the rights and welfare of subjects [3]. Trainees may not have been aware of these stipulations, and we hypothesized that some residents experienced discomfort with the abbreviated, one-page consent process. In our survey, 9/50 (18%) reported they were somewhat or very uncomfortable with the modified consent process. When asked about specific ethical concerns, none (0%) reported that patient autonomy and patient welfare were not respected at all through the modified consent process. However, 12/50 (24%) of participants reported that patients received too little information about their participation in the trial.

Overall, our results demonstrate that GME trainees can effectively implement a PCT, but some trainees may not support integrating pragmatic research into their clinical training. The survey did not include a formal qualitative component, but we hypothesize several explanations based on our experience and unsolicited comments in the survey. Some residents felt the additional research duties on top of clinical responsibilities were burdensome to an already busy clinical service and outside the mission of their training. Some reported that the alteration of informed consent resulted in inadequate transparency about the research study and that there was substantial variability in its delivery among so many providers. Unfortunately, these concerns may be in tension, as providing more information to participants would likely result in increased time and workload on trainees.

It is an ongoing project of pragmatic research to balance the protection of research participants with the need for better evidence generation [4,5]. While optional training in ethics has been integrated into some GME programs, it is unclear whether trainees receive any instruction on how to navigate their dual responsibilities of patient care and clinical research when involved in pragmatic trials [6]. Future studies could explore whether providing residents with a targeted curriculum of pragmatic research ethics or the opportunity to participate in study design alleviates their concerns about the ethics or workload of conducting a PCT. For example, if residents were involved in the writing of the standardized script for consent, they could point out any perceived issues about the minimal risk or patient welfare determinations made by the IRB. Trainees have the potential to advance pragmatic research, and their perspectives should be used to address the operational and ethical challenges of these studies.

## Supporting information

Newton et al. supplementary material Newton et al. supplementary material

## Supplementary material

The supplementary material for this article can be found at https://doi.org/10.1017/cts.2026.10687.
