# Supplementary material for: Embedding pragmatic clinical trials in graduate medical education: Lessons from implementation
Source: J Clin Transl Sci. 2026 Jan 28;10(1):e32. doi: 10.1017/cts.2026.10687 (PMC12936871; doi:10.1017/cts.2026.10687)
Supplement: Newton et al. supplementary material [file S2059866126106876sup001.pdf]

Resident Perspectives on Participation in a Pragmatic Randomized Trial

You have been identified as a current or former resident who participated in enrollment of patients into the IMpact of PerioperAtive KeTamine on Enhanced Recovery after Abdominal Surgery (IMPAKT ERAS) trial at VUMC.

This trial utilized a modified consent process completed by anesthesiology residents alongside their clinical evaluation and regional anesthesia consent.

We are interested in exploring resident experiences with this consent process. This information will help guide further research and study design of pragmatic trials at VUMC. Your participation is voluntary, your responses will be anonymous, and any decision to participate (or not participate) will not impact your in-training progress.

		Absolutely Inappropriate	Somewhat Inappropriate	Neutral	Somewhat Appropriate	Absolutely Appropriate
1	How appropriate or inappropriate was the use of the resident workforce to consent and enroll patients for this pragmatic clinical trial?	○	○	○	○	○

		Very Uncomfortable	Somewhat Uncomfortable	Neutral	Somewhat Comfortable	Very Comfortable
2	How comfortable or uncomfortable were you with the modified consent process?	○	○	○	○	○

		Too Little	Right Amount	Too Much
3	How would you characterize the amount of information patients received about their participation in this trial?	○	○	○

		Too Little	Right Amount	Too Much
4	How would you characterize the amount of instruction you received on how to consent patients for this trial?	○	○	○

		Not respected at all	Somewhat respected	Completely respected
5	To what degree was patient autonomy respected through the modified consent process?	○	○	○

		Not respected at all	Somewhat respected	Completely respected
6	To what degree was patient welfare respected through the modified consent process?	○	○	○

		Never	Infrequently	Neutral	Frequently	Every time
7	How often did you use the model script as part of the modified consent process?	○	○	○	○	○

		Never	Infrequently	Neutral	Frequently	Every time
8						

	How often did you deviate from the model script as part of the modified consent process?	○	○	○	○	○

		Would not recommend	Neutral	Would recommend
9	Would you recommend or not recommend that this resident-led modified consent process be used to answer future research questions in your field?	○	○	○

10 Please comment or elaborate on any responses above.
